# Sternal Wound Infection after Cardiac Surgery: Management and Outcome

**DOI:** 10.1371/journal.pone.0139122

**Published:** 2015-09-30

**Authors:** Marie Dubert, Annabelle Pourbaix, Soleiman Alkhoder, Guillaume Mabileau, François-Xavier Lescure, Walid Ghodhbane, Sabine Belorgey, Christophe Rioux, Laurence Armand-Lefèvre, Michel Wolff, Richard Raffoul, Patrick Nataf, Yazdan Yazdanpanah, Jean-Christophe Lucet

**Affiliations:** 1 Infectious Diseases Department, Bichat-Claude Bernard Hospital, Assistance-Publique Hôpitaux de Paris, Paris, France; 2 Cardiac Surgery Department, Bichat-Claude Bernard Hospital, Assistance-Publique Hôpitaux de Paris, Paris, France; 3 IAME, UMR 1137, INSERM, F-75018, Paris, France; Univ Paris Diderot, Sorbonne Paris Cité, F-75018, Paris, France; 4 Infection Control Unit, Bichat-Claude Bernard Hospital, Assistance-Publique Hôpitaux de Paris, Paris, France; 5 Bacteriology Laboratory, Bichat-Claude Bernard Hospital, Assistance-Publique Hôpitaux de Paris, Paris, France; 6 Medical Intensive Care Unit, Bichat-Claude Bernard Hospital, Assistance-Publique Hôpitaux de Paris, Paris, France; Peking University People Hospital, CHINA

## Abstract

**Background:**

Sternal Wound Infection (SWI) is a severe complication after cardiac surgery. Debridement associated with primary closure using Redon drains (RD) is an effective treatment, but data on RD management and antibiotic treatment are scarce.

**Methods:**

We performed a single-center analysis of consecutive patients who were re-operated for SWI between 01/2009 and 12/2012. All patients underwent a closed drainage with RD (CDRD). Patients with endocarditis or those who died within the first 45 days were excluded from management analysis. RD fluid was cultured twice weekly. Variables recorded were clinical and biological data at SWI diagnosis, severity of SWI based on criteria for mediastinitis as defined by the Centers for Disease Control (CDC), antibiotic therapy, RD management and patient’s outcome.

**Results:**

160 patients developed SWI, 102 (64%) fulfilled CDC criteria (CDC+) and 58 (36%) did not (CDC- SWI). Initial antibiotic treatment and surgical management were similar in CDC+ and CDC- SWI. Patients with CDC+ SWI had a longer duration of antibiotic therapy and a mortality rate of 17% as compared to 3% in patients with CDC- SWI (p = 0.025). Rates of superinfection (10% and 9%) and need for second reoperation (12% and 17%) were similar. Failure (death or need for another reoperation) was associated with female gender, higher EuroScore for prediction of operative mortality, and stay in the ICU.

**Conclusion:**

In patients with SWI, initial one-stage surgical debridement with CDRD is associated with favorable outcomes. CDC+ and CDC- SWI received essentially the same management, but CDC+ SWI has a more severe outcome.

## Introduction

Sternal wound infection (SWI) still is a frequent complication after sternotomy for cardiac surgery, with an incidence of 0.5 to 3% [1–4]. Variations in definition partly explain the large range of infection rates. The most commonly used definition is mediastinitis as defined by the Centers for Disease Control and Prevention (CDC)[5]. Other authors proposed a broader definition, including infection of presternal tissue requiring reoperation, sternal osteomyelitis and mediastinitis [6].

The incidence of mediastinitis has barely declined over the last decades. This may be explained by the fact that cardiac surgery is more frequently performed in patients with higher risk of infection, despite improvement in preventive measures. SWI leads to prolonged hospital stay and is responsible for in-hospital mortality rates of 8–28% [7–10].

In infected patients, multiple publications have described and compared the surgical treatment, using aggressive surgical debridement followed by open packing, irrigation and drainage, closed drainage with Redon drains (CDRD) or vacuum-assisted closure (VAC) [11–15]. However, there is no consensus on the optimal surgical management of SWI. Surprisingly, the medical counterpart of surgical treatment has been rarely described and evaluated [10,11]. Because mediastinitis is an infection that involves large spaces and sternal bone, the medical treatment frequently translates from the treatment of osteo-articular and hip or knee infected prosthesis. Although antibiotic therapy is not standardized, treatment duration of 3–6 weeks is frequently advocated, without clear supporting data [10,11].

We thus retrospectively investigated a large cohort of patient presenting with SWI, and uniformly receiving the same surgical treatment. Our objective was firstly to describe the various infections grouped under the term of SWI (presentation, medical management and outcome); secondly to evaluate the impact of CDC classification on the management and in particular medical treatment; and thirdly, to assess the outcome and variables associated with poor outcome.

## Patients and methods

### Definition

Mediastinitis was defined according to the CDC criteria, with either (i) organisms cultured from mediastinal tissue or fluid obtained during a surgical operation or needle aspiration, or (ii) evidence of mediastinitis seen during a surgical operation or histopathologic examination, or (iii) at least one of the following with no other recognized cause: fever (>38.8°C), chest pain or sternal instability and at least one of the following: a/ purulent discharge from mediastinal area, b/ organisms cultured from blood or discharged from mediastinal area, or c/ mediastinal widening on x-ray [5]. Therefore, SWI without sternal reopening requiring reoperation with the presence of bacteremia was classified as mediastinitis according to the CDC definition.

### Study design and patients

We conducted a retrospective study in Bichat—Claude Bernard Hospital, where about 1,100 cardiac surgical procedures with median sternotomy and extracorporeal circulation are performed each year. All patients with a SWI between January 2009 and December 2012 that required reoperation were included.

During the first surgical procedure, the major preventive measures were as follows: patients with a preoperative nasal carriage of *Staphylococcus aureus* (*S*. *aureus*), were decolonized using mupirocin and antiseptic soap; all patients received antibiotic prophylaxis with cefamandole, unless known or suspected carriage of *Staphylococcus aureus* resistant to methicillin (MRSA), in which case vancomycin plus gentamicin was used. Coronary artery bypass grafting (CABG) was performed using both internal thoracic arteries (ITA) in most cases, whereas single ITA grafting was performed only if CABG complemented a predominant valvular surgery.

All files were analyzed until three months after initial surgery for identification of re-operated patients. Briefly, the infection control team recorded all reoperations after the first surgical procedure. Early reoperations for hemorrhage or pericardial effusion were discarded. All other reoperations were reviewed and clinical data such as fever (>38.0°C); presence of sepsis, severe sepsis or shock [16]; local inflammatory signs including purulent discharge, scar disjunction, sternal instability, sternal pain; biological data; bacteriological data, and operative findings were collected. Bacteriological data included results of transcutaneous needle aspiration of the sternum before reoperation, bacteriological culture during reoperation, and the first culture of the closed RD after operation.

Each suspicion was cross-validated with senior cardiac surgeons to identify SWI with or without sternal reopening [17,18].

For the sake of the study, we included for initial description all patients with SWI, including those who died during the first 45 days after the first surgical procedure (the maximal duration of antibiotic therapy) and those with associated infectious endocarditis. However, these patients were excluded from the analysis of medical and surgical management because loss to follow-up or specific antimicrobial therapy driven by this infection.

### Initial medical and surgical management

All patients with SWI underwent surgical debridement, thorough removal of necrosis material and primary CDRD. According to the operative findings, sternum was reopened or not, sternal wires were tightened and skin was meticulously closed to ensure the absence of leaks. The decision to reopen the sternum depended on the presence of pus originating from the retrosternal area. Two to 10 RD were placed in the infected area and connected to numbered plastic bottles with a high negative pressure of 600 mmHg. The fluid collected from all plastic bottles was cultured twice a week. RD were progressively removed after at least three consecutive negative samples and at least two weeks of drainage.

Empirical intravenous antibiotic therapy was started just before reoperation, based if possible on direct examination or results of preoperative samples from the sternal wound. In the absence of preoperative microbiological documentation, a triple antibiotic therapy was initiated including a broad-spectrum beta-lactam combined with vancomycin and an aminoglycoside.

After obtaining the results of perioperative cultures, the infectious disease physician (IDP) from the antibiotic stewardship team advocated the antibiotic therapy. The duration of antibiotic treatment, duration of the antimicrobial association if any, intravenous duration, and overall duration, were determined in each case by the IDP, according to microorganisms cultured from the SWI and perioperative findings: whether the sternum was reopened or not, defining the mediastinal involvement. The common practice was to treat mediastinal infection for 6 weeks, including 3 weeks of intravenous therapy and superficial infection for 3 to 4 weeks, including 10 to 15 days of intravenous therapy. Duration of an antimicrobial association also depended on the involved microorganism.

We collected the following parameters: demographic characteristics, including age, gender and unit(s) of hospital stay; EuroScore, designed to predict post-operative mortality [19]; the type of initial intervention; clinical presentation at infection onset; results of microbiological cultures; bacteremia, defined as at least one positive blood culture, except for skin commensals requiring at least two positive blood cultures; management of RD with date of first negative culture and date of removal; antibiotic therapy, including single therapy or association, the duration of intravenous therapy and the total duration of antibiotic therapy; stay in the ICU after the first 1–3 post-operative days in the cardiac surgical ICU; and date of death or hospital discharge. Information was obtained for all patients for at least 6 months.

The following complications were collected: RD colonization, defined by the presence of a new microorganism cultured from RD collecting bottles without any sign or symptom of infection; superinfection if the presence of a new microorganism required a change in the antibiotic therapy, with or without a subsequent surgical procedure; persistent infection if the initial pathogen remained despite appropriate antibiotic, mandating a new surgery. The need for new reoperation was also collected, together with the type of surgical treatment after debridement, either open packing, a new CDRD or VAC.

Because the study was observational and did not impact on patient’s care, and because the study was retrospective, informed consent was waived by the local ethical Committee. The study protocol was approved by the ethic committee “CEERB Paris Nord” (Institutional Review Board-IRB 00006477- of HUPNVS, Paris 7 University, AP-HP).

### Statistical analysis

Quantitative variables were expressed as median with interquartile range (IQR) and qualitative variables were expressed as percentages. We compared patients within and without mediastinitis according to the CDC definition. Statistical tests included Mood’s median test and Chi-squared test, using Yates’ correction method when applicable.

To assess variables associated with outcome in infected patients, we developed a composite failure score, which included either death from any cause within the first six months after reoperation for SWI or the need for a reoperation after the first surgical procedure for SWI. Patients with both second reoperation then death were censored at the time of reoperation. Patients who did not meet the composite failure score were censored at six month or at the date they were last seen alive at the hospital after six month. A Cox proportional hazard model was used to assess variables associated with failure in bivariate and multivariate analysis for variables that were significantly associated with failure in bivariate analysis, according to the backward stepwise method. Statistical analysis was performed using R software (version 2.15.2).

## Results

### Population description

**Fig 1:**
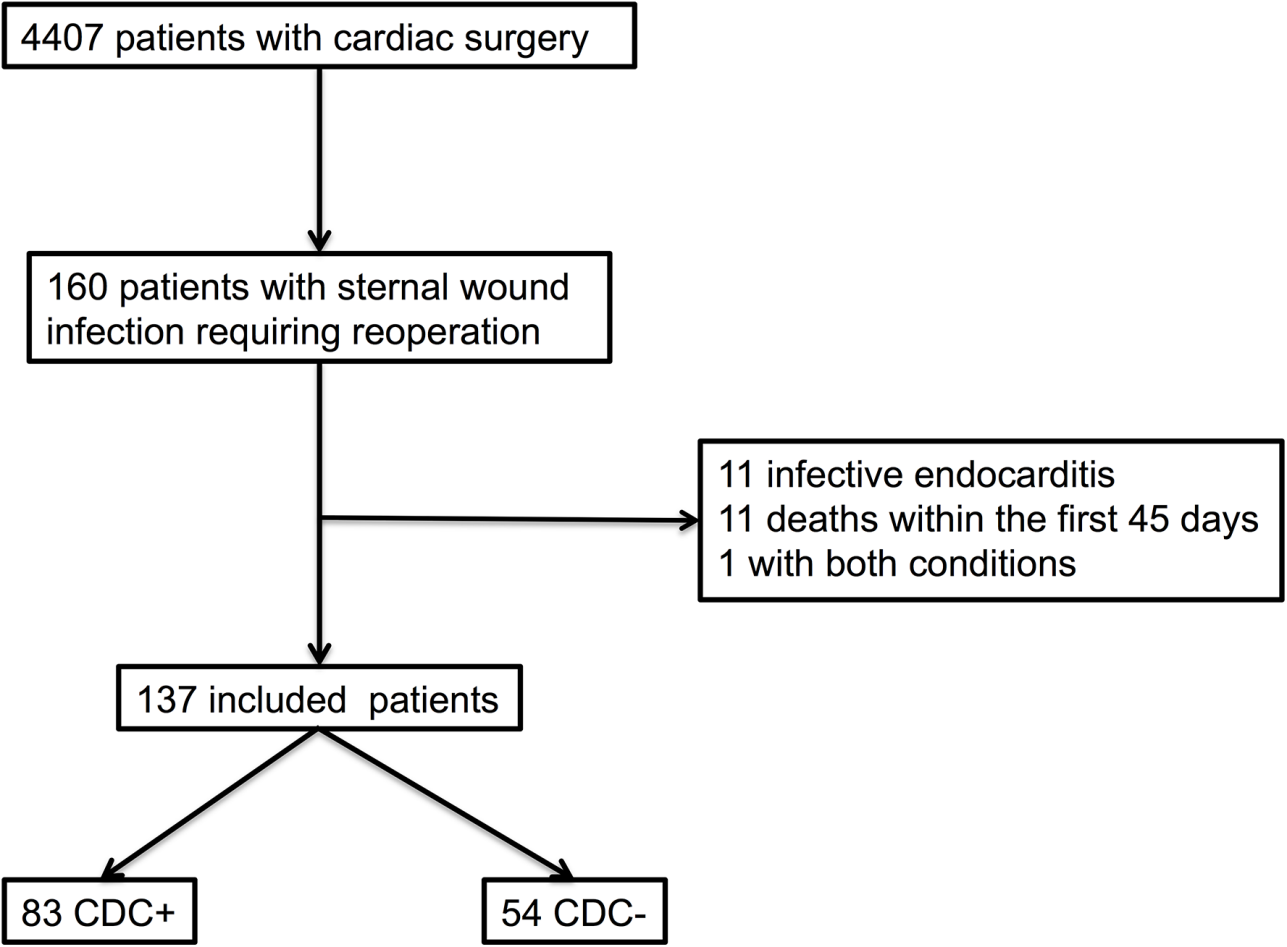
Flow chart of Patients with Sternal Wound Infection Requiring Reoperation after Cardiac Surgery.

During the study period, 160 patients developed a SWI requiring reoperation, of which 102 (64%) were mediastinitis according to the CDC definition (CDC+) and 58 without the CDC criteria of mediastinitis (CDC-) (Fig 1).

The proportion of mediastinitis and of all SWI requiring reoperation in the population undergoing cardiac surgical procedures with median sternotomy and extracorporeal circulation (ECC) was 2.3% (102/4407) and 3.6% (160/4407), respectively. Among the 102 mediastinitis, 54 infections involved the mediastinal area requiring sternum reopening, and 48 met the CDC definition because of bacteremia in patients not requiring sternum reopening. The median age of the 160 patients was 69.3 years (IQR [60–77]) without difference between CDC+ patients and CDC- patients, and 96 (60%) were male. Initial cardiac surgery was CABG in 107/160 (67%) patients, valve replacement in 25/160 (16%) patients, a combined surgery in 22/160 (14%) patients, a cardiac transplantation in two and another surgery in four patients. The overall 6-month mortality was 19/160 (12%).

For this population, clinical presentation is displayed in Table 1. The median time from initial surgery to reoperation was significantly shorter in CDC+ patients than in CDC- patients, 17 days versus 21 days, p = 0.01. A total of 109 patients (68%) were reoperated within the first 30 postoperative days, of whom 69 (43%) were with CDC+ SWI. Of the 160 infected patients, 57 (36%) were referred to the ICU at any time after the typical first 1–3 days in the cardiovascular ICU, among which 25/57 (44%) were already in ICU before SWI. Stay in the ICU was more frequent in CDC+ patients (44/102, 43%) than in CDC- patients (13/58, 22%, p<0.01). Fever and severe sepsis or shocks were significantly more frequent in CDC+ than in CDC- patients (p<0.001).

**Table 1:** Initial Presentation of 160 Patients with Sternal Wound Infection Requiring Reoperation after Cardiac Surgery.

	CDC+ (n = 102)	CDC- (n = 58)	p-value
Age (years), median [IQR]	70.5 [60–78]	66 [61–76]	0.20
Male, n (%)	67 (66%)	29 (50%)	0.05
Surgical procedure, n (%)			0.001*
CABG	58 (57%)	49 (84%)	
Valular repair	22 (21%)	3 (5%)	
Both procedures	18 (18%)	4 (7%)	
Other	4 (4%)	2 (4%)	
EuroScore, median [IQR]	8 [6–11]	8 [6–12]	0.88
Local inflammatory signs, n (%)	82 (80%)	51 (88%)	0.22
Fever, n (%)	46 (51%)	8 (16%)	<0.001
Severe sepsis or shock, n (%)	27 (32%)	3 (6%)	<0.001
Time from the initial surgery to surgery for SWI (days), median [IQR]	17 [12–25]	21 [16–30]	0.01
Sternum reopening, n (%)	54 (53%)	0 (0%)	<0.001
Total stay in the in ICU, n (%)	44 (43%)	13 (22%)	<0.01
Stay in the ICU before SWI, n (%)	21 (20%)	4 (7%)	0.02
Transfer to the ICU because of SWI, n (%)	23 (23%)	9 (16%)	0.23

* The two last modalities were brought together for the test because the conditions of validity for a Chi-square test at 3 degrees of freedom were not verified

Among 160 patients with SWI, 153 (96%) had a microbiologically documented infection, of which 46 (29%) were polymicrobial. Main microorganisms are displayed in Table 2.

**Table 2:** Microbiological Data in 160 Patients with Sternal Wound Infection Requiring Reoperation after Cardiac Surgery, n (%).

	CDC+ (n = 102)	CDC- (n = 58)	p-value
Coagulase-negative staphylococci (CoNS)	42 (31%)	30 (42%)	0.12
*Staphylococcus aureus* (MRSA)	32 (4) (23%)	8 (3) (11%)	0.03
Enterobacteriacae	35 (26%)	16 (22%)	0.58
Other microorganism	27 (20%)	18 (25%)	0.39
No documented infection	2 (2%)	5 (9%)	0.12
Polymicrobial infection	28 (27%)	18 (31%)	0.63
Associated bacteremia	54 (53%)	0 (0%)	<0.001

Abbreviation: MRSA, Methicillin-resistant staphylococcus aureus; CoNS, Coagulase-negative staphylococci

### Management

Eleven patients (8%) had a suspected or proven associated endocarditis; 11 patients died within the 45 days after reoperation for SWI, and one patient had endocarditis and died. Thus our analysis of SWI management was limited to 137 patients, including 83 CDC+ patients and 54 CDC- patients.

Among the 137 included patients, the median [IQR] time from reoperation to negative results of microbiological sampling of all RD and to RD removal was 6 days [4–10] and 20 days [18–23], respectively, without difference between CDC+ and CDC- SWI (Table 3).

**Table 3:** Surgical and Medical Management in 137 Patients with Sternal Wound Infection Requiring Reoperation after Cardiac Surgery according to the Definition.

	CDC+ (n = 83)	CDC- (n = 54)	p-value
Number of Redon drains (days), median [IQR]	5 [3–6]	3 [2–4]	<0.004
Time to culture-negative RD (days), median [IQR]	6 [3–10]	7 [5–12]	0.29
Time to RD removal (days), median [IQR]	20 [18–24]	20 [17–22]	0.68
Duration of intravenous antibiotic treatment (days), median [IQR]	20 [13–27]	17 [7–21]	0.09
Duration of antibiotic association (days), median [IQR]	29 [18–42]	19 [5–26]	<0.001
Overall duration of antibiotic treatment (days), median [IQR]	39 [28–44]	31 [22–38]	0.04
Secondary RD colonization n (%)	28 (34%)	15 (28%)	0.46
Superinfection, n (%)	10 (13%)	5 (9%)	0.49
2nd reoperation for persistent or superinfection, n (%)	12 (14%)	10 (19%)	0.53
Length of hospital stay (days), median [IQR]	25 [20–37]	24 [21–34]	0.33

There was a longer overall median duration of antimicrobial treatment in CDC+ patients (39 days) than in CDC- patients (31 days, p = 0.04). The median duration of intravenous antibiotic treatment was 20 and 17 days in patients with CDC+ and CDC- SWI (p = 0.09). The median duration of antibiotic association was significantly longer in patients with CDC+ SWI than in patients with CDC- SWI (p<0.001) (Table 3).

Of the 137 followed patients, 43 (31%) developed at least one RD colonization after a median time from reoperation of 13 days, without difference between CDC+ and CDC- SWI. Two main colonizing microorganisms were identified, coagulase-negative staphylococci (CoNS) (n = 41, 68%) and Enterobacteriacae (n = 14, 23%). Colonization was more frequent in patients staying in the ICU (21/43, 49%) than in patients outside the ICU (22/94, 23%, p<0.001). Fifteen patients (11%) developed superinfection, without difference between CDC+ and CDC- SWI. The main pathogens (n = 21) were CoNS (n = 10, 48%) and Enterobacteriacae (n = 9, 43%). Among the 15 patients with RD superinfection, the median overall duration of antibiotic therapy was longer than in patients without superinfection, 43 and 32 days, respectively, p<0.01.

Twenty-two patients (16%) underwent at least another reoperation, without difference between CDC+ and CDC- SWI. A total of 29 new reoperations were performed, once in 16 patients, twice in 5 patients, and one patient underwent more than 2 reoperations. Seventeen of the 29 reoperations were for the treatment of a persistent SWI (n = 10) and/or a superinfection (n = 13). The first new reoperation required sternum reopening for debridement in 8/22 patients, and the wound was closed on CDRD in 11 patients and on VAC in 8 patients. Two patients required a muscular plasty for final closure of the sternal wound at their third and fourth reoperation.

### Outcome

Of the 160 patients with SWI, 19 (12%) died within the first 6 months after reoperation. The death rate was 17/102 (17%) in patients with CDC+ SWI and 2/58 (3%) in patients with CDC- SWI (p = 0.03). Twenty-five of the 160 patients (16%) underwent at least another reoperation of the sternal wound, of whom 8 died.

Therefore, 36 patients (23%) fulfilled the criteria of the composite failure score. The univariate and multivariate analysis are presented in Table 4. In the multivariate Cox model analysis, the following variables were significantly associated with failure: higher EuroScore, female sex and stay in the ICU.

**Table 4:** Variables Associated with Failure (n = 36), Cox Proportional Hazard Model.

	Population	Failure	HR[IC 95%]	P	Adjusted HR [IC 95%]	p-value
**Age** (years), median [IQR]	69 [60–77]	70 [63–78]	1.01 [0.98–1.03]	0.70		
**Body mass index,** n (%)						
26–29	39 (24%)	5 (14%)	Ref			
< 26	40 (25%)	11 (32%)	3.28 [1.06–10.17]	0.04		
> 30	81 (51%)	20 (56%)	2.56 [0.88–7.49]	0.09		
**EuroScore,** median [IQR]	8 [6–11]	11 [9–16]	1.19 [1.09–1.29]	<0.001	1.11 [1.02–1.22]	0.02
**Female gender,** n (%)	64 (40%)	24 (68%)	3.33 [1.67–6.66]	<0.001	3.01 [1.50–6.05]	<0.01
**Surgical procedure,** n (%)						
CABG	107 (67%)	23 (64%)	Ref	-		
Valvular repair	25 (15%)	7 (19%)	1.30 [0.56–3.03]	0,54		
Both procedures	22 (14%)	6 (17%)	1.30 [0.53–3.20]	0.56		
Other	6 (4%)	0 (0%)	0.00 [0.00-inf]	0.99		
**Type of SWI,** n (%)						
CDC-	58 (36%)	12 (33%)	Ref			
CDC+ (mediastinitis)	102 (64%)	24 (66%)	1.18 [0.59–2.38]	0.62		
**Causative pathogen,** n (%)						
*S*. *aureus*	40 (25%)	4 (11%)	Ref	-		
CoNS	56 (35%)	8 (22%)	1.44 [0.43–4.78]	0.55		
Enterobacteriacae	50 (31%)	17 (47%)	3.90 [1.31–11.58]	0.01		
Other	14 (9%)	7 (19%)	6.31 [1.84–21.57]	<0.01		
**Stay in the ICU,** n (%)						
No	103 (64%)	12 (12%)	Ref	-	Ref	-
Before SWI	25 (16%)	11 (44%)	4.80 [2.11–10.9]	<0.001	3.86 [1.65–9.05]	<0.01
After SWI	32 (20%)	13 (41%)	4.17 [1.90–9.14]	<0.001	2.95 [1.32–6.57]	<0.01

Abbreviation: HR, Hazard ratio; BMI, Body Mass Index; SWI, CABG, Coronary Artery Bypass Grafting; SWI, Sternal Wound Infection; *S*. *aureus*, *Staphylococcus aureus*, CoNS, coagulase-negative staphylococci; CDC, Centers for Diseases control and Prevention; ICU, Intensive care unit

## Discussion

In this large single-centre cohort of patients undergoing major cardiac surgery, the mortality associated with SWI requiring reoperation was 12%. SWI requiring a reoperation resulted in a prolonged antibiotic treatment and duration of hospital stay. The patients with SWI but without the CDC criteria for mediastinitis (CDC- SWI) had a similar medical and surgical management and similar overall duration of hospital stay as patients with CDC-defined mediastinitis, but an improved outcome. Failure was associated with higher preoperative risk of death, stay in the ICU and female gender.

In the present study, the rate of SWI varied according to the definition used, from 1.6% with CDC-defined mediastinitis occurring within 30 post-operative days to 3.6% if the definition included all reoperations for SWI. As a consequence, benchmarking can be difficult. The CDC definition could be used to assess the outcome and the impact of SWI. Differentiating superficial SWI requiring reoperation from the CDC-defined mediastinitis however may be difficult since surgical and medical management are essentially similar. Therefore, as others [6], we suggest using a broader definition when assessing SWI management. Extending the definition of mediastinitis to all SWIs requiring reoperation could allow easier classification of SWIs, based on a reproducible and robust definition, and thus facilitate a long-term and simple surveillance. Definition based on prognosis criteria and severity of the SWI rather than patho-physiology may be more clinically relevant.

CDC-defined mediastinitis occurred earlier and with different microorganisms than CDC- SWI. Contrasting with other series, *S*. *aureus* was found in only 25% of patients with SWI, presumably due to perioperative nasal and skin decolonization. However, *S*. *aureus* was more frequently found in CDC+ SWI than in CDC- SWI, and was more frequently associated with bacteraemia. By contrast, CoNS was the most frequent pathogen isolated in CDC- SWI, infection which occurred later than mediastinitis. These latter infections may correspond to delayed healing and mechanical dehiscence, with secondary superinfection of the wound, and less severe outcome [20]. Polymicrobial infection was observed one fourth of infected patients, usually with the association of bacteria from the digestive tract, enterobacteriacae and *Enteroccus spp*. This could be ascribable to nasal decolonization, therefore protecting the patient from *S*. *aureus* infection, and replacement by bacteria from the digestive tract.

Morbidity associated with CDC+ and CDC- SWI was similar, as shown by the total duration of hospital stay and the need for a second reoperation. This similar duration of hospital stay was essentially driven by a similar duration of RD in both groups, whereas the duration of antibiotic treatment was shorter in patients with CDC- SWI. By contrast, crude mortality was significantly higher in CDC+ SWI than in CDC- SWI, because of the higher preoperative risk of mortality, and presumably by the severity of infection, as shown by a higher frequency of severe sepsis and shock and more frequent transfer to an ICU. The 12% overall mortality, 3% in CDC- SWI and 17% in CDC-defined mediastinitis, however was in accordance with recent publications [8,15,21].

All 160 patients with SWI were uniformly treated with one-stage surgical debridement and primary closure with CDRD. The 16% failure rate of this technique is similar to other surgical techniques [22]. In addition, the long 20-day median duration of CDRD requires careful supervision and exposes the wound to superinfection. The safety of shortening the CDRD duration requires further investigation.

The median duration of antibiotic therapy was significantly longer in CDC+ SWI (5.5 weeks) than in CDC- SWI (4.5 weeks). This difference is not clinically relevant since SWI requiring reoperation is usually considered to be associated with sternal bone involvement requiring prolonged antibiotic treatment. Despite a different prognosis for death, CDC+ and CDC- SWI have a similar morbidity and management. Based on our experience, antibiotic therapy should not last more than 3 weeks for patient with CDC-SWI, with a maximum of 10 days of intravenous therapy. As for prosthetic osteoarticular infection, there is an urgent need for defining the best duration of antibiotic therapy in every situation.

We developed a composite failure score and survival analysis using a Cox proportional model to account for the complex relationship between death from SWI, death from another cause and the need for a second reoperation. As a result, variables associated with the composite score reflected (i) either the underlying disease or complicated course after the surgical procedure with the EuroScore and stay in the ICU before SWI; (ii) the impact of SWI on outcome reflected by the need for transfer to the ICU because of SWI; (iii) the risk factor for new reoperation, with female gender [8,23]. Indeed, female gender may be associated with SWI, presumably because of tension of the breast on skin wound.

The major strengths of our study are the large cohort of patients and the homogeneity of SWI management, driven together by the cardiac surgeons and the IDPs. To the best of our knowledge this is the first description of the complete combined medical and surgical SWI management after cardiac surgery. There are however several limitations. Firstly, contrary to the uniform surgical management, the antibiotic duration was not protocolized, thus we cannot draw firm conclusion about the optimal management and duration of antibiotic treatment. Secondly, detailed information regarding causes of death was not available, consequently we can not attribute the death to SWI. At last, we stopped the surveillance at 6 months after initial reoperation. Therefore, the long-term impact of SWI cannot be assessed, especially with regard to persistent SWI and the need for further reoperation. However, the 6-month surveillance reasonably allows for assessing early failure and death.

In conclusion, the present study confirms that SWI requiring reoperation after cardiac surgery is associated with favorable outcomes when using one-stage surgical debridement and CDRD. Failure was associated with higher EuroScore, female sex and stay in ICU. Additional informations remain to be obtained regarding the optimal duration of Redon drainage and of antibiotic treatment.

## Supporting Information

S1 Dataset (XLS)
